# Manipulation of Light Signal Transduction Factors as a Means of Modifying Steroidal Glycoalkaloids Accumulation in Tomato Leaves

**DOI:** 10.3389/fpls.2018.00437

**Published:** 2018-04-12

**Authors:** Cui-cui Wang, Lan-huan Meng, Ying Gao, Donald Grierson, Da-qi Fu

**Affiliations:** ^1^Fruit Biology Laboratory, College of Food Science and Nutritional Engineering, China Agricultural University, Beijing, China; ^2^School of Biosciences, University of Nottingham, Nottingham, United Kingdom

**Keywords:** glycoalkaloids, light-stimulated, tomato leaves, ELONGATED HYPOCOTYL5, Phytochrome interacting factors

## Abstract

Steroidal glycoalkaloids (SGAs) are cholesterol-derived specialized metabolites produced by Solanaceous plant species. They contribute to pathogen defense but are considered as anti-nutritional compounds and toxic to humans. Although the genes involved in the SGA biosynthetic pathway have been successfully cloned and identified, transcription factors regulating this pathway are still poorly understood. We report that silencing tomato light signal transduction transcription factors ELONGATED HYPOCOTYL 5 (*SlHY5*) and PHYTOCHROME INTERACTING FACTOR3 (*SlPIF3*), by virus-induced gene silencing (VIGS), altered glycoalkaloids levels in tomato leaves compared to control plant. Electrophoretic mobility shift assay (EMSA) and Chromatin immunoprecipitation (ChIP) analysis confirmed that SlHY5 and SlPIF3 bind to the promoter of target genes of GLYCOALKALOID METABOLISM (*GAME1, GAME4, GAME17*), affecting the steady-state concentrations of transcripts coding for SGA pathway enzymes. The results indicate that light-signaling transcription factors HY5 and *PIF3* regulate the abundance of SGAs by modulating the transcript levels of these *GAME* genes. This insight into the regulation of SGA biosynthesis can be used for manipulating the level of these metabolites in crops.

## Introduction

Steroidal glycoalkaloids (SGAs) are nitrogen-containing compounds produced primarily by Liliaceous (*Veratrum californicum*) (Augustin et al., 2015) and Solanaceous species such as potato *(Solanum tuberosum)*, tomato (*Solanum lycopersicum*), and eggplant (*Solanum melongena)* (Heftmann, 1983). SGAs are stored in all plant tissues, including roots, flowers, leaves, and fruits (Friedman and Dao, 1992; Friedman, 2006; Kozukue et al., 2008; Iijima et al., 2013). Although SGAs act as phytoanticipins, providing the plant with a pre-existing defense against a broad range of pathogens (Milner et al., 2011), some are considered as anti-nutritional substances in the diet due to their toxic effects (Roddick, 1996). SGAs can cause gastro-intestinal and neurological disorders, and may be lethal to humans when present at high concentrations (Roddick, 1989). About 100 steroidal alkaloids (SAs) have been found in the different tissues and development stages of tomato (Moco et al., 2006; Mintz-Oron et al., 2008a; Schwahn et al., 2014). In green tomato tissues, the principal SGAs are α-tomatine and dehydrotomatine, while esculeosides are predominant in the red ripe fruits (Fujiwara et al., 2004; Moco et al., 2007; Yamanaka et al., 2009).

Currently, research on SGAs is focused mainly on the elucidation of their structures and composition in different plant species and unraveling their biosynthetic pathway (Itkin et al., 2013; Schwahn et al., 2014). SGAs are biosynthesized from a common precursor, cholesterol (Eich, 2008). Cholesterol undergoes several hydroxylation, oxidation, transamination and glycosylation steps during the biosynthesis of SGAs. Three genes (*SGT1, SGT2*, and *SGT3*) responsible for glycosylating SGA have been identified in potato (Moehs et al., 1997; McCue et al., 2006, 2007). In tomato, GLYCOALKALOID METABOLISM 1 (*GAME1*) glycosyltransferase, the homolog of SGT1 in potato, adds a galactose to the aglycone tomatidine molecule (Itkin et al., 2011). Recently, Itkin identified other genes encoding enzymes catalyzing the conversion of cholesterol to SGAs. The *GLYCOALKALOID METABOLISM* (*GAME*) genes, located close to each other on the genome and organized in a metabolic gene cluster, take part in the primary pathway synthesizing SGAs. In tomato, genes encoding cytochrome P450s (*GAME7, GAME8a, GAME8b*, and *GAME6* located in a cluster on chromosome 7), *GAME4* (on chromosome 12) and a dioxygenase (*GAME11* on chromosome 7) are involved in the hydroxylation and oxidation of the cholesterol skeleton, while a transaminase protein (*GAME12* on chromosome 12) incorporates the nitrogen atom into the SA aglycone. Finally, the glycosyltransferases (*GAME1, GAME17, GAME18*, and *GAME2* on chromosome 7) add the sugar moieties to tomatidine to form tomatine (Itkin et al., 2013).

The structural genes involved in the biosynthesis of SGAs have been researched intensively, but the transcriptional regulation of SGA biosynthesis is unclear. Some transcription factors (TFs) of the APETALA2/Ethylene Response Factors (AP2/ERF) family regulate the biosynthesis of terpenoid indole alkaloids (TIAs) in some plants. The AP2/ERF TF ORCA3, induced by jasmonate, regulates the biosynthesis of TIAs in *Catharanthus roseus* (van der Fits and Memelink, 2000; Zhang et al., 2011; De Geyter et al., 2012). Close homologs of ORCA3, present in the *NIC2* locus, regulate nicotine biosynthesis in the tobacco leaf (Hibi et al., 1994). *ERF189* and *ERF221/ORC1*, also located in the *NIC2* locus, regulate nicotine biosynthesis in tobacco (Sutter et al., 2005; Shoji et al., 2010; Todd et al., 2010; De Boer et al., 2011) and the *NIC2* locus comprises at least seven ERF TFs that regulate the expression of structural genes in the biosynthesis of nicotine (Shoji et al., 2010). In Solanaceous plants, *GLYCOALKALOID METABOLISM 9* (*GAME9*), an APETALA2/Ethylene Response Factor, regulates the biosynthesis of SGAs and several upstream mevalonate and cholesterol precursor pathway genes (Cárdenas et al., 2016).

Light and other environmental factors play key roles in plant growth and development. Previous studies have shown that phytochromes and cryptochromes are involved in the crosstalk between the mevalonate (MVA) and methylerythritol 4-phosphate (MEP) pathways (Lichtenthaler, 1999), which affects the synthesis of cholesterol, the precursor of SGAs. In potato, the specific SGAs produced in response to light vary with different regions of the emission spectrum and duration of irradiation (1 week and 14 days) (Percival, 1999; Zrust et al., 2001).

Light quality and quantity are perceived by various photoreceptors and signal transduction involves the modulation of downstream TFs such as PHYTOCHROME INTERACTING FACTORS (PIFs) and ELONGATED HYPOCOTYL 5 (HY5) (Lau and Deng, 2010). The allosteric conformational change of phytochromes from an inactive Pr form to a biologically active Pfr form is followed by nuclear translocation and direct interaction with PIFs, which results in phosphorylation and degradation of PIFs (Leivar and Monte, 2014). HY5, on the other hand, has been shown to be the key target of the CONSTRUCTIVE PHOTOMORPHOGENIC1/SUPPRESSOR OF phyA-105 (COP1/SPA) complex (Osterlund et al., 2000) and a recent study proposed that the binding of phytochrome B to SPA inhibits the activity of COP1 (Sheerin et al., 2015).

Many studies have demonstrated that HY5 regulates cholesterol synthesis. HY5, a basic leucine zipper (bZIP) TF, integrates signals from photoreceptors to suppress the activity of 3-hydroxy-3-methylglutaryl CoA reductase (HMGR; EC 1.1.1.34) in the MVA pathway (Rodríguez-Concepción et al., 2004). Lee et al. (2007) reported that in the cholesterol biosynthesis pathway, the 2C-methyl-D-erythritol 2, 4-cyclodiphosphate synthase gene (At1g63970) in the upstream MEP pathway plus two terpene synthases genes (At3g14520, At3g25830) and one geranylgeranyl pyrophosphate synthase gene (GGPS, At4g38460) contain HY5 binding sites in their promoters, shown by chromatin-immunoprecipitation using DNA chip hybridization (ChIP-chip). It has also been shown that HY5 could bind to over 9,000 genes, over 1,100 were detectably affected in their expression, and these included a wide range of genes encoding proteins involved in light-signaling components.

HY5 promotes photomorphogenesis in the light, however, PIFs, which are basic helix-loop-helix (bHLH) TFs, are active and regulate gene expression to promote skotomorphogenesis in seedlings in the dark (Leivar et al., 2008). The PIFs that have been examined so far (PIF1, PIF3, PIF4, PIF5, and PIF7) also bind to a core promoter DNA G-box motif (CACGTG) in a sequence-specific manner (Toledo-Ortiz et al., 2003; De Lucas et al., 2008). Each specific light response is regulated by one, two, or three PIFs. For example, PIF1 promotes cotyledon appression and inhibits seed germination (Oh et al., 2004; Shin et al., 2009). PIF1 and PIF3 both promote hypocotyl negative gravitropism and suppress chlorophyll biosynthesis (Huq et al., 2004; Stephenson et al., 2009; Kim et al., 2011). PIF4 and PIF5 are related to plant rhythmic growth (Nozue et al., 2011; Nomoto et al., 2012). PIF4, PIF5, and PIF7 promote shade avoidance (Lorrain et al., 2009; Li et al., 2012). In addition to light responses, PIF4 regulates high temperature-induced hypocotyl elongation while PIF3 is responsible for regulating ethylene-induced hypocotyl elongation (Koini et al., 2009; Zhong et al., 2012). Among the phytochrome-interacting proteins, PIF3 is the most extensively characterized to date. Using yeast two-hybrid screening, PIF3 was identified originally as a phytochrome-interacting protein (Ni et al., 1998). In a related study (Halliday et al., 1999), *photocurrent 1* (*poc1*), an *Arabidopsis* mutant perturbed in phytochrome signaling due to a T-DNA insertion in the promoter of PIF3, had higher PIF3 transcript levels than the wild type under red light, and down-regulated PIF3 expression when grown in the dark. Biochemical analysis indicated that the Pfr form of phytochrome interacts reversibly with PIF3 bound to the G-box element of various promoters (Ni et al., 1999; Martínez-García et al., 2000), such as those in CIRCADIAN CLOCK ASSOCIATED 1 (*CCA1*) and LATE ELONGATED HYPOCOTYL (*LHY*). The reduced photo-inducibility of these two genes in PIF3 antisense transgenic plants confirms the view that phytochrome signals are targeted directly to the promoters of light-inducible genes through PIF3. Aghamirzaie et al. (2017) reported that Acyl-CoA oxidase (At4g16760) in the upstream MVA pathway, and sterol 4-alpha-methyl-oxidase 2-1 (At1g07420) contain PIF3 binding sites in their promoters by ChIP-chip.

The role of light signal transduction factors in the biosynthesis of glycoalkaloids remains to be elucidated. In our study, tomato plants placed in the dark showed a decrease in the expression of SGA-related genes. Virus-induced gene silencing (VIGS) showed that HY5 and PIF3 regulate SGA biosynthesis. Furthermore, chromatin immunoprecipitation, in combination with quantitative PCR (ChIP-PCR) and promoter binding assays demonstrated that HY5 and PIF3 could directly bind to the promoters of downstream target genes. These results not only provide insights into the transcriptional regulation of SGAs in Solanaceous plants, but also provide a theoretical basis for the removal of these anti-nutritional compounds and for ensuring the food safety of crops.

## Materials and methods

### Preparation of plant materials

Wild-type cultivar Micro-Tom tomato plants were grown in a climate-controlled greenhouse at 22°C (night) and 25°C (day) with an 8:16 h dark: light photoperiod, with supplementary illumination with LED fluorescent lamps, luminous efficiency 90 lm/W. Tomato seeds were germinated in the soil in plastic pots. For the dark treatment, 3-weeks-old tomato plants were transferred to a darkroom in complete darkness for 8 days.

### Vector construction

cDNA regions for gene silencing were selected to avoid off-targeting by the VIGS design tool (http://solgenomics.net/tools/vigs). The required restriction enzyme site was added at the end of the primer. Three fragments (500 bp *SlPDS*, 538 bp *SlHY5*, and 400 bp *SlPIF3)* were amplified from tomato cv. Micro-Tom cDNA using PCR (Liu et al., 2002). pTRV2*-SlPDS* and pTRV2-*SlHY5* constructs were generated by inserting each PCR fragment into the pTRV2 vector digested with *Kpn* I and *Xho* I, and pTRV2-*SlPIF3* with *EcoR* I and *BamH* I. Oligonucleotide primers used are listed in Table S1.

### *Agro*-infiltration

VIGS was performed using Tobacco Rattle Virus (TRV) (Liu et al., 2002; Fu et al., 2005). *Agrobacterium* strain *GV3101* containing the TRV-VIGS vectors was grown at 28°C in Luria–Bertani (LB) liquid medium (pH 5.6) containing 10 mM 2-(N-morpholino)- ethanesulfonic acid (MES) and 20 μM acetosyringone and the antibiotics kanamycin, gentamycin and rifampicin. After shaking for 18 h, the cells were harvested and resuspended in the *Agrobacterium* inoculation buffer (10 mM MgCl_2_, 200 μM acetosyringone, 10 mM MES, pH 5.6) to a final OD_600_ of 2.0. Resuspensions of pTRV1 and pTRV2 or its derivative vectors were mixed at a 1:1 ratio and incubated at room temperature for 3 h. The *Agrobacterium* was infiltrated into tomato seedlings with a 1 ml needleless syringe (**Figure 2B**). Seedlings infiltrated with pTRV1 and pTRV2 were used as controls. Each inoculation was carried out three times with 20 different plants each time. After 3 weeks, leaves were collected and stored at −80°C.

### Isolation of total RNA and reverse transcription

Total RNA was isolated from leaves of silenced and non-silenced (infiltrated with empty vectors pTRV1 and pTRV2) plants grown for 30 days, using TRIzol reagent (Invitrogen). RNA quantity and purity were measured using a NAS-99 spectrophotometer (ATCGene, NJ, USA). RNA integrity was verified by 1.5% agar gel electrophoresis. Genomic DNA was removed from extracted total RNA by digestion with DNase I (Takara, China). One microgram of total RNA was reverse-transcribed using the PrimeScript 1st Strand cDNA Synthesis Kit (Takara) with oligo dT primer in a 20 μL system following the manufacturer's instructions.

### Quantitative real-time PCR analysis

The gene-specific oligonucleotide primers used for quantitative real-time PCR (qRT-PCR) are listed in Table S1. The length of the qRT-PCR products ranged from 80 to 300 bp. Quality and specificity of each pair of primers were checked by melting curves and product resequencing. Quantitative reverse transcription-PCR (qRT-PCR) was conducted using SYBR Green PCR Master Mix with a real-time PCR System CFX96 (Bio-Rad, CA, USA). The reactions were performed with the following cycling profile: 95°C for 10 min, followed by 39 cycles of 95°C for 15 s and 60°C for 30 s. Melting curve analysis was performed to verify the specificity of the amplification for each primer pair. Fluorescence changes of SYBR Green were monitored automatically in each cycle and the threshold cycle (Ct) over the background was calculated for each reaction. *Actin* gene (Solyc03g078400) results were used to normalize samples. The relative expression levels were calculated using the 2^−ΔΔ*Ct*^ method (Livak and Schmittgen, 2001).

### Isolation of SGA biosynthetic gene promoters

Genomic DNA was extracted from cv. Micro-Tom tomato leaves using a DNAsecure plant kit (TIANGEN, Beijing) according to the manufacturer's protocol. Promoter regions of genes involved in the biosynthesis of SGAs, including *GAME1, GAME2, GAME4, GAME6, GAME11, GAME12, GAME17*, and *GAME18*, were identified from NCBI (https://www.ncbi.nlm.nih.gov/) (Table S3). Primers used for ChIP-PCR are listed in Table S2. Conserved promoter *cis*-element motifs were predicted using the Motif Scan database (https://myhits.isb-sib.ch/cgi-bin/motif_scan/) and Plant-CARE database (http://bioinformatics.psb.ugent.be/webtools/plantcare/html/).

### Preparation of plant extracts and metabolite analysis

Extraction of SGAs was carried out as described previously (Itkin et al., 2011) with three biological replicates. Briefly, cv. Micro-Tom tomato leaves (300 mg) were frozen in liquid nitrogen and ground to a fine powder using a mortar and pestle. Then, the powder was treated with 900 μl 80% methanol: water (v/v) containing 0.1% formic acid (the solid: liquid ratio was kept at 1:3 w/v). The mixture was vortexed for 30 s, sonicated for 30 min at room temperature, vortexed again for 30 s, centrifuged (20,000 g, 10 min) and filtered through a 0.22 μm aperture polytetrafluoroethylene membrane filter (Acrodisc CR 13 mm; PALL). The filtrate was transferred to an autosampler glass vial with a 100 μl conical glass insert and analyzed by HPLC–MS/MS according to Itkin et al. (2013). Tomatine was identified by comparison of its retention time and mass spectrum with those generated for the corresponding standard compounds (α-tomatine [Apin Chemicals]) analyzed using the same equipment. The following modifications were used: tomato extracts were analyzed in MRM positive mode using an HPLC-MS/MS (Agilent) equipped with an Acquity C18 column and Triple Quadrupole MS detector ((Mintz-Oron et al., 2008)a). The absorption spectrum maximum is 270 nm. The mobile phases were water with 0.1% (v/v) formic acid (A) and acetonitrile (B), using a gradient elution of 5–100% B during 0–30 min (linear gradient). The MS parameters were: capillary−4.0 kV, Cone−60V, collision energy−60eV. MRM transitions were set as 1,034.5 > 416.3 and 1,034.5 > 578.3. The second transition trace was used for α-tomatine quantification.

### Protein expression and electrophoretic mobility shift assay (EMSA)

The coding region of HY5 (1–477 bp) and PIF3 (1,162–1,854 bp, covering the protein functional region) were PCR-amplified and cloned into pGEX-4T-1 (Amersham Biosciences) to fuse in frame with GST. The constructed vector was transformed into *Escherichia coli* strain BM Rosetta (DE3). EMSA assays were performed as previously described (Ye et al., 2016). After growing to saturation, the transformed cells were treated with 0.5 mM isopropyl β-D-1-thiogalactopyranoside (IPTG) and incubated at 28°C for 4 h to induce fusion protein expression. The recombinant protein was purified using a Pierce GST spin purification kit (Thermo Scientific). Resulting proteins were checked for size by SDS-PAGE using 250 kD protein marker (Bio-Rad) and Coomassie Brilliant Blue staining. Protein concentration was determined using an RC/DC protein assay kit based on the Lowry assay (Bio-Rad). Synthetic oligonucleotides (50 bp) for the *GAME* promoter were biotinylated. EMSA was performed using biotin-labeled probes and the Lightshift Chemiluminescent EMSA kit (Thermo Scientific) following the manufacturer's protocol (Shan et al., 2014). Briefly, the assay mixtures were incubated for 25 min at 22°C. All of the reaction products were analyzed by 6% native polyacrylamide gel electrophoresis. After cross-linking, the membrane was incubated in the blocking buffer for 15 min, and then transferred to conjugate/blocking buffer. Finally, the membrane was washed three times, for 5 min each time. Biotin-labeled DNA was detected by the chemiluminescence method on a ChemiDoc™ MP Imaging System (Bio-Rad). The primers used in the EMSA assay are listed in supporting information Table S1.

### HY5/PIF3 antibody and western blot analysis

For HY5 polyclonal antibody production, the peptide C^*^ AGTQRKRGRSPADKEN was synthesized. The C^*^ (cysteine) was added for coupling. Polyclonal PIF3 antibodies were generated by cloning the *PIF3* (433–1,254 bp) complementary cDNA into the pET-30a expression vector (Novagen). The peptides and His_6_-tagged PIF3 protein were injected into rabbits four times at 2-week intervals (Huada Corporation, Beijing).

All cv. Micro-Tom tomato seedlings grown in the greenhouse for 40–50 days, and then the protein was extracted. Leaves (100 mg) were mixed, frozen and ground into powder, then 500 μl denaturing buffer (100 mM NaH_2_PO_4_, 10 mM Tris–HCl pH 8.0, 8 M urea, 1 mM PMSF, and 1 complete protease inhibitor mixture (Roche) were added. Extracts were centrifuged at 16,000 g for 10 min at 4°C, and protein concentration in the supernatants was quantified by the Bradford assay. Equal volumes of the extract were loaded on the gels and western blots were performed (Osterlund et al., 2000). Five micrograms total protein extracted from the tomato leaves were separated by SDS-PAGE, and the proteins in the gel transferred onto PVDF membranes (0.22 μm pore size; Immobilon-P Membrane®, Bio-Rad, USA) by electroblotting at a constant current of 90 mA for 1 h. After blotting, the nitrocellulose membrane was cut into strips to test the individual sera. 5% non-fat dried milk in PBS containing 0.05% Tween 20 (PBST) was used to block non-specific binding sites for 1 h at room temperature with constant agitation. After PBST washing, the strips were incubated with the tested antibody, anti-HY5, and anti-PIF3, each diluted (1:1,000) in 1.5% milk PBST overnight at 4°C with constant agitation. Following incubation and washing six times in PBST, the strips were incubated with anti-rabbit IgG peroxidase conjugate (peroxidase-conjugated goat anti-rabbit IgG (Sigma-Aldrich Corporation, USA) diluted 1:1,000 in milk PBST for 1 h at room temperature with constant agitation. After three 15 min PBST washes, the strips were incubated with a peroxidase substrate (3, 3′-N-diaminobenzidine tetrahydrochloride substrate solution; Sigma, USA) to visualize the protein bands (Ye et al., 2016). The reproducibility of the result was confirmed by three independent experiments (*n* = 3) (Santana et al., 2017).

### Chromatin immunoprecipitation

Antibodies of HY5 and PIF3 were prepared by the HuaDa Corporation (Beijing). HY5 and PIF3 antibodies were attained by three stages including protein purification, immunization and antibody purification, and final detection. Tomato leaves (20 g) from the greenhouse were fixed in formaldehyde and the nuclei isolated (Ricardi et al., 2010). After sonication, the suspension was transferred to a 2 ml tube, using 1/10 of the supernatant as input. 25 μl Dynabeads (Pro A for rabbit) were washed twice with low salt wash buffer (600 μl) in a Protein Lobind tube (1.5 ml). The beads were resuspended in 200 μl low salt wash buffer and 2 μl purified antibody (1.5 mg/ml) or 100 μl positive serum added. The beads-antibody-mix was incubated at 4°C on a rotator for 1–2 h. The tubes were snap spun, the beads recovered on a magnetic stand at 4°C, washed with low-TE buffer once and antibody-beads added to the sample and incubated overnight. The supernatant was removed and the beads resuspended and washed several times; 5 μl NEB proteinase K was added and incubated at 55°C for 30 min and then reverse crosslinked at 65°C overnight. To the reverse crosslinked product in a 1.5 ml tube was added an equal volume of chloroform to extract the DNA. The sample was shaken for 1 min and then centrifuged at 12,000 g for 10 min at 4°C. The supernatant was removed to a new low binding tube very carefully. Two microliters Roche Glycogen (20 mg/ml) were first added to the supernate, followed by 1/10 V_supernate_ of NaAc [3 M,pH 5.2] and 3V_supernate_ of ethanol, incubate at −20°C for 15 min, spun at 12,000 g for 30 min at 4°C. The supernatant was discarded and the precipitate washed with 75% ethanol and centrifuged, the ethanol supernatant discarded and the pellet dissolved in 20–30 μl TE buffer. Finally, the reverse crosslink product was purified. DNA isolated from the precipitated chromatin was analyzed to determine which DNA fragments were present in the precipitate. Quantitative PCR for the detection of immunoprecipitated DNA was used to normalize data. The primer sets used met specific criteria (Wong and Medrano, 2005; Kubista et al., 2006), in order to obtain high quality qPCR data (Table S2). The normalization methods for ChIP analysis took account of background subtraction (Mutskov and Felsenfeld, 2004), percentage of input (Nagaki et al., 2003) and fold-enrichment (Tariq et al., 2003).

### Dual-luciferase transient expression assay

Dual-luciferase transient expression assays (DLR) in *Nicotiana benthamiana* were performed as described previously (Ye et al., 2016). Briefly, the promoters of *SlGAME1, SlGAME4*, and *SlGAME17* and mutant promoters were transferred individually to the reporter vector (pGreenII 0800-LUC) of the dual luciferase reporter system to drive the expression of LUC, while *SlHY5* and *SlPIF3* were introduce into the pEAQ effector vectors driven by the CaMV35S promoter (**Figure 8A**). *Agrobacterium* EHA105 containing constructed reporter and effector plasmids separately were used for transient expression in tobacco leaves, with a mixing ratio of *Agrobacterium* is 9:1. The dual-luciferase assay kit (Promega) was used to analyze the transient expression in tobacco leaves 3 days after infiltration. Absolute LUC/REN was measured in a Luminoskan Ascent Microplate Luminometer (Thermo Scientific) according to the manufacturer's instructions, with a 5 s delay and 15 s integrated measurements. The transactivation ability of *SlHY5* and *SlPIF3* are indicated by the ratio of LUC to REN. At least six biological replicates were assayed for each combination.

## Results

### Dark treatment reduces the expression of *game* genes in tomato leaves

**Figure 1:**
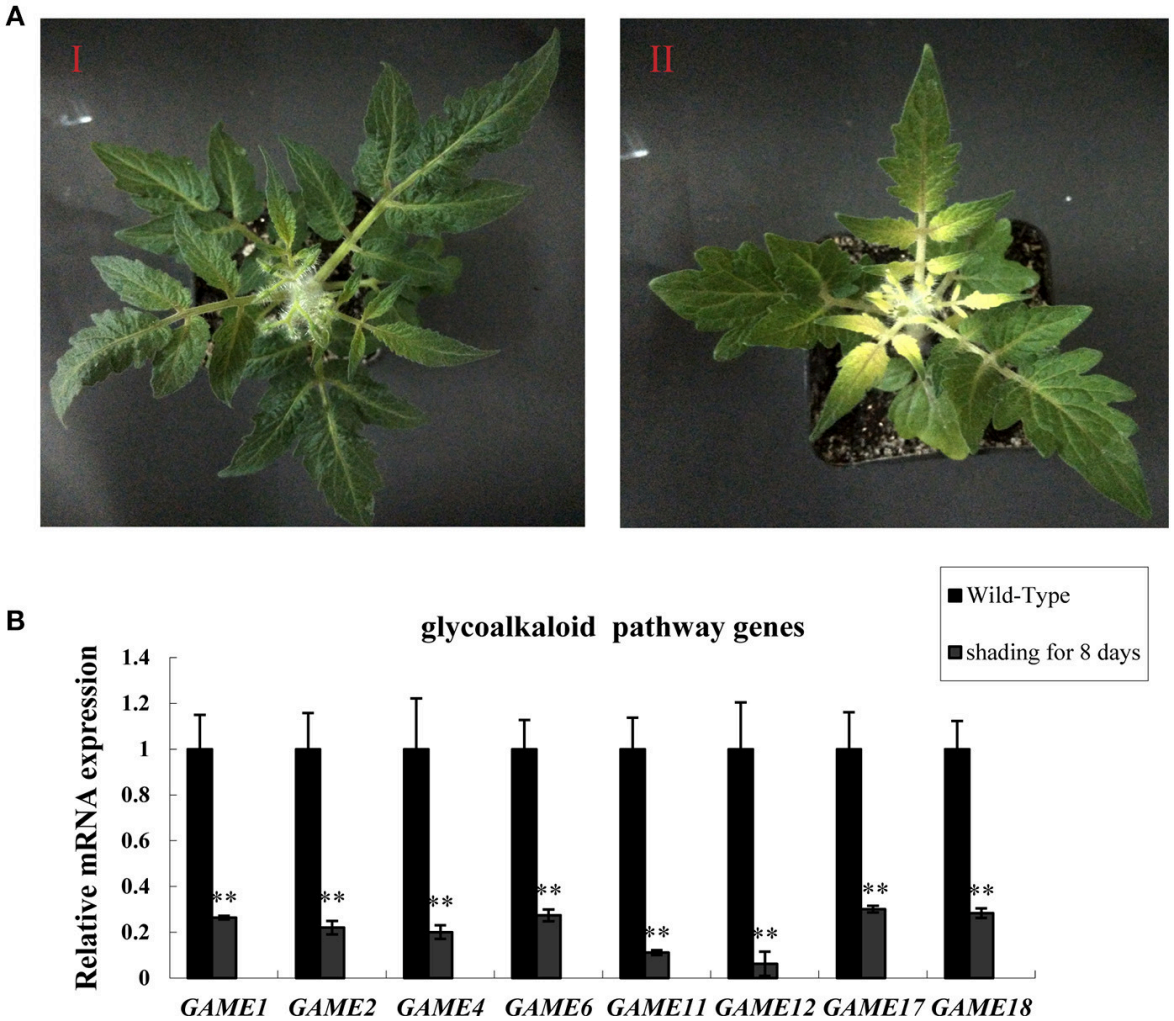
Dark treatment inhibits SGA gene expression in tomato leaves. **(A)** Phenotypes of tomato leaves kept in the dark. (I) Wild-type tomato leaves from plants in the light (II) Tomato leaves in the dark for 8 days. **(B)** *GAME* mRNA relative expression, measured by qRT-PCR. Data are the mean ± standard deviation of three biological replicates. Student's *t-*test indicates significant changes from control plants: ^*^P-value < 0.05; ^**^P-value < 0.01. Primers designed for Real-time quantitative RT-PCR are listed in Table S1.

To investigate the effect of light on the expression of SGA-related genes in leaves, tomato plants that had been grown for 20 days in a 16:8 light: dark photoperiod were placed in darkness for 8 days (Figure 1A). Leaves kept in the dark showed reduced expression of glycoalkaloid biosynthesis-related genes in all three samples compared to the leaves of plants in the light: dark photoperiod (Figure 1B). The reduction in SGAs mRNA levels in the dark ranged from ~77–90%, suggesting that the biosynthesis of SGAs may be enhanced by increased expression of glycoalkaloids biosynthesis-related genes in the light.

### VIGS of *SlHY5* and *SlPIF3* in tomato leaves

**Figure 2:**
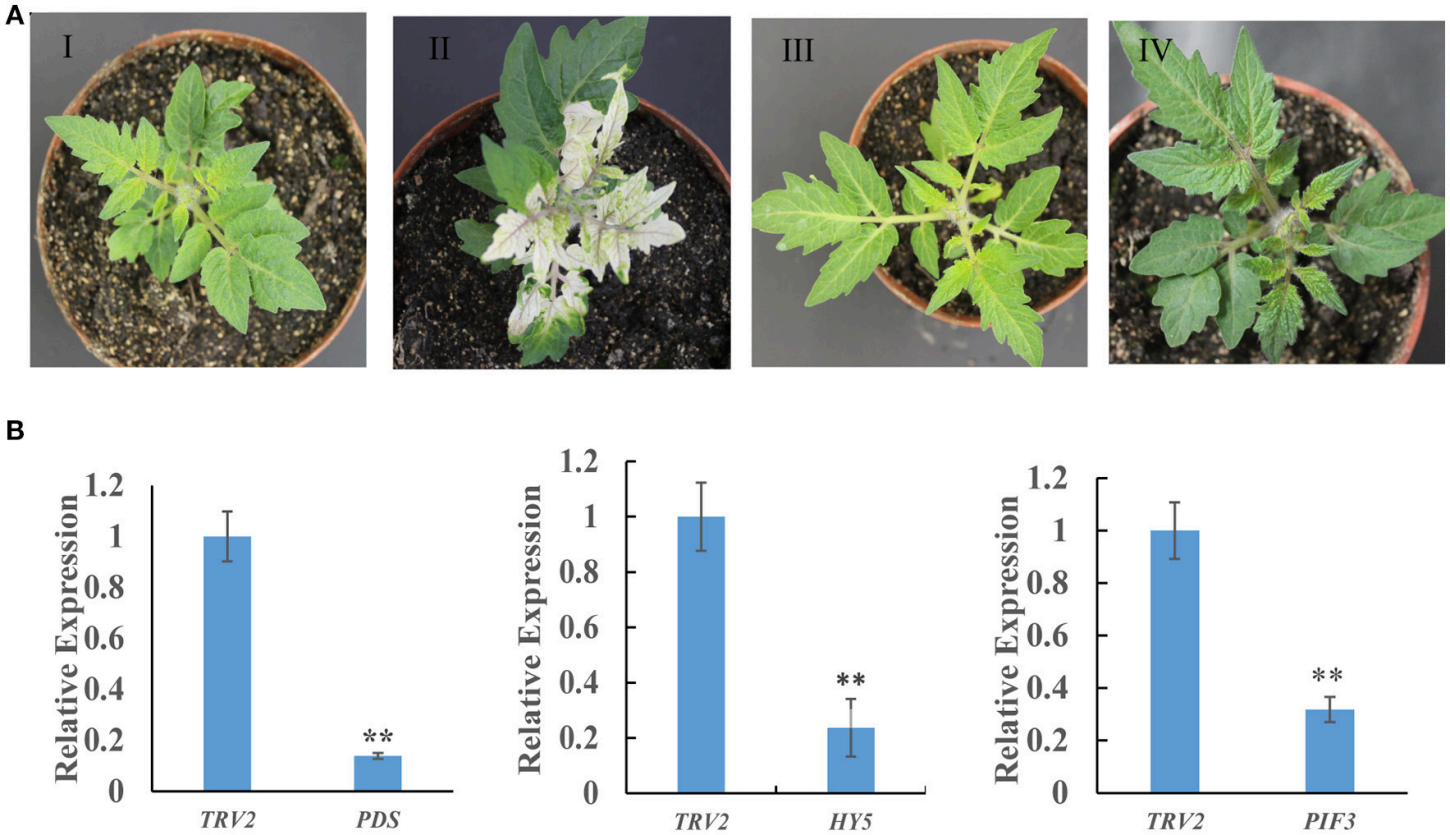
Tobacco rattle virus-mediated silencing of *PDS, HY5*, and *PIF3* in tomato leaves. **(A)** Phenotypes of tomato leaves following infiltration with different VIGS constructs: I, Empty vector; II, *TRV-PDS*; III, *TRV-HY5*; IV, *TRV-PIF3*. **(B)** Quantitative RT-PCR measurement of *PDS, HY5*, and *PIF3* mRNA. Data are the mean ± standard deviation of three biological replicates. PDS, phytoene desaturase; HY5, transcription factor LONG HYPOCOTYL5; PIF3, Phytochrome Interacting Factor 3. Student's *t*-test was used to assess whether the silenced plants differed significantly from wild-type plants: ^*^P-value < 0.05; ^**^P-value < 0.01. Oligonucleotides used are shown in Table S1.

To investigate whether the biosynthesis of SGAs is regulated by light signaling, a VIGS approach was used to silence the *SlHY5* and *SlPIF3* genes. *SlPDS* was used as a positive selection procedure to identify the *SlPDS* silenced regions for tissue sampling. *SlHY5* leaves of TRV-VIGS silenced plants exhibited no typical viral infection phenotype compared to control plants. In contrast, plants in which *SlPIF3* expression was silenced produced dark green leaves (Figure 2A). Analysis of all leaves showed the presence of both the TRV1 and TRV2 genomes fragments (Fu et al., 2005). Further investigation confirmed that VIGS-treated leaves showed a reduction in *SlPDS, SlHY5*, and *SlPIF3* transcripts ranging from ~78 to 85% in all three samples compared to leaves of TRV2-infected control plants (Figure 2B).

### Altering *SlHY5* and *SlPIF3* expression impacts the levels of major SGAs

**Figure 3:**
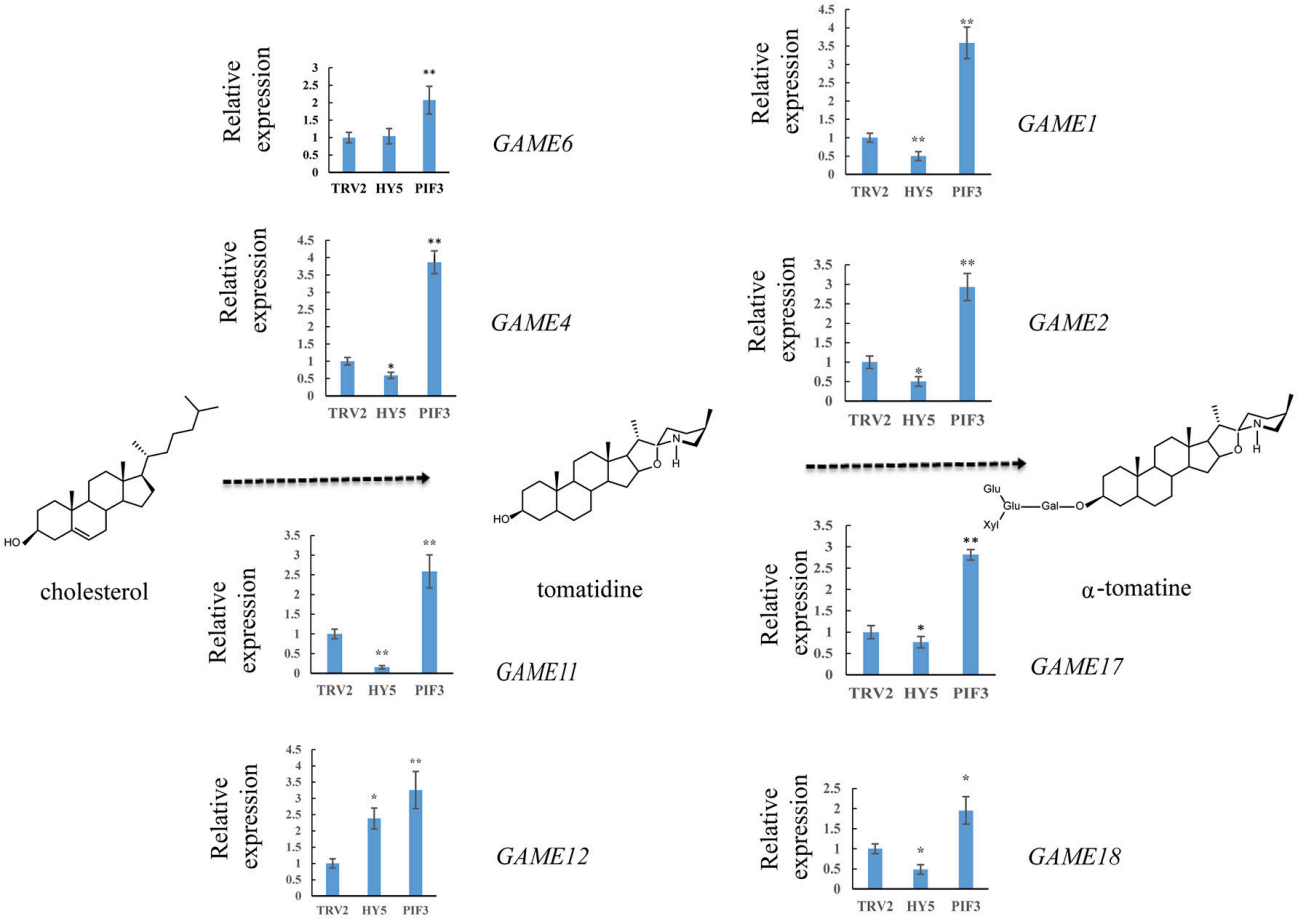
Relative expression levels of glycoalkaloid pathway genes in leaves following infiltration with different constructs. A schematic view of the SGA biosynthetic pathways. Dashed arrows represent multiple biosynthetic reactions. Graphs next to each gene name show expression levels determined by qRT-PCR, normalized to the *Actin* housekeeping gene and to the mock-infected control. Data are mean ± standard deviation of three biological replicates. Asterisks indicate a significant difference from the control according to Student's *t*-test (^**^P < 0.01 and ^*^P < 0.05).

**Figure 4:**
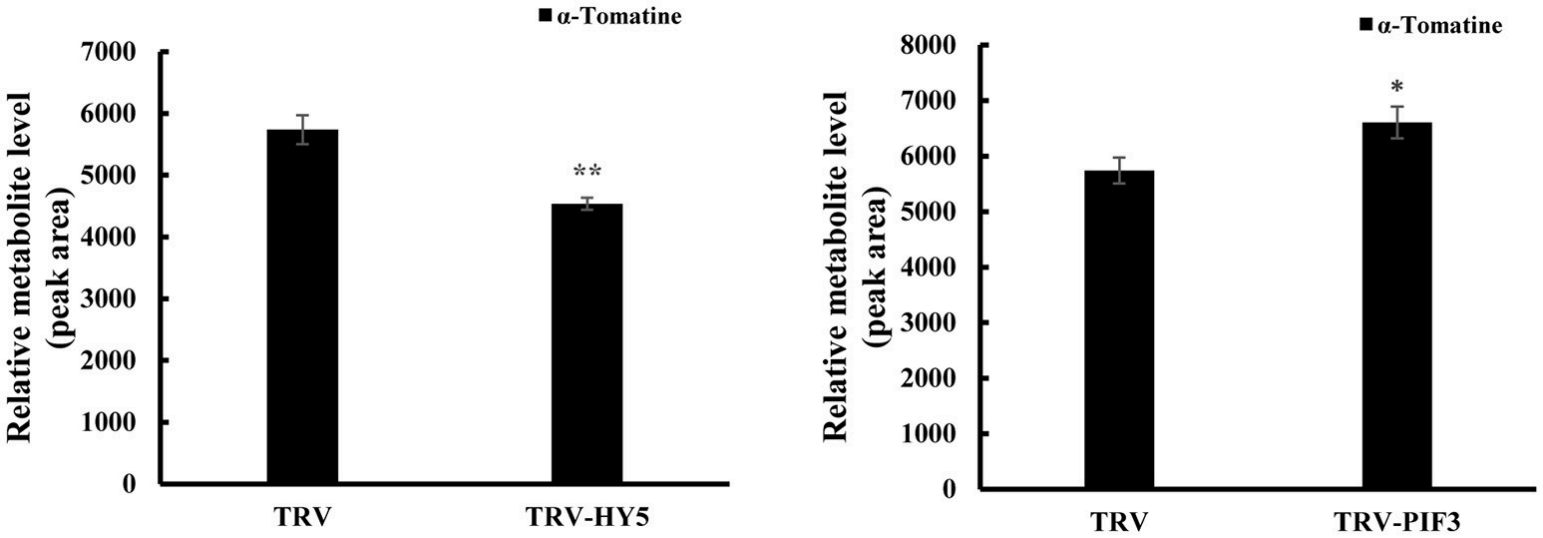
Changes in HY5 and PIF3 expression in tomato leaves results in altered levels of the predominant SGAs. Levels of a-tomatine in leaves determined by UPLC-MS/MS. Tomatine was identified by comparison of its retention time and mass spectrum to those generated for authentic standards analyzed on the same instrument. Relative metabolite levels are expressed as ratios of peak areas. Values represent means ± standard deviation (*n* = 3). Student's *t*-test was used to assess whether the results with the virus-induced gene silencing plants differed significantly from those for TRV-infected plants (^*^P < 0.05; ^**^P < 0.01).

To investigate whether light signal transduction factors influence glycoalkaloid biosynthesis, we studied the effect of inhibiting expression of *SlHY5* using VIGS mediated by TRV (Liu et al., 2002). In the *SlHY5*-silenced tomato leaves, the *GAME* genes responsible for the biosynthesis of tomatidenol (*GAME11* and *GAME4*) and its subsequent glycosylation (*GAME1, GAME2, GAME17*, and *GAME18*) were significantly downregulated, however, the expression of *GAME12* was increased (Figure 3). In leaves of *SlPIF3*-silenced tomato plants, the relative levels of *GAME1, GAME4*, and *GAME12* transcripts increased 3.2- to 4.1-fold, 3.4- to 4.3-fold, and 3.0- to 4.2-fold, respectively, compared to leaves of TRV-infected plants (Figure 3). Similarly, there was an increase in *GAME2* (2.3- to 3.0-fold), *GAME6* (1.4- to 2.3-fold), *GAME11* (2.3- to 2.8-fold), *GAME17* (2.7- to 3.0-fold) and *GAME18* (1.6- to 2.1-fold) compared to the control plants (Figure 3). The glycoalkaloids contituents were analyzed by HPLC-MS/MS, which showed a 21% reduction in tomatine in TRV-*SlHY5*-infected leaves compared to TRV-infected leaves, and a 16% increase in tomatine in leaves in which *PIF3* was silenced (Figure 4).

### HY5 and PIF3 specifically bind to the promoters of SGAs biosynthesis-related genes

**Figure 5:**
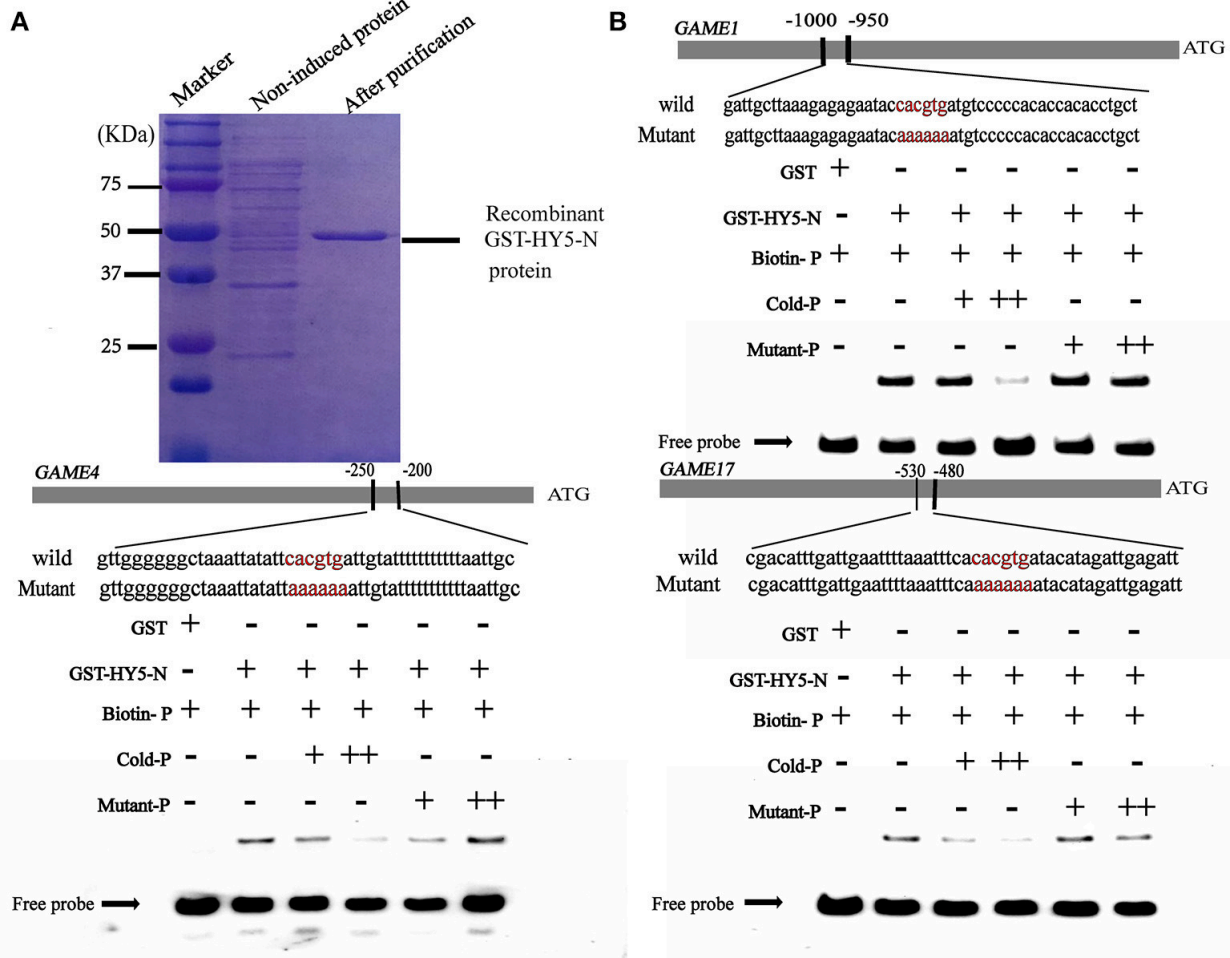
Electrophoretic mobility shift assay (EMSA) showing the association of *Sl*HY5 with promoters of SGA biosynthetic genes *SlGAME1, SlGAME4*, and *SlGAME17*. **(A)** SDS–PAGE gel stained with Coomassie blue showing affinity purification of the recombinant *Sl*HY5 protein used for the EMSA. **(B)** *Sl*HY5 binds directly to the G-box (CACGTG) element of the promoters of *SlGAME1, SlGAME4*, and *SlGAME17*. Biotin-labeled DNA probes for the promoters or mutated probes were incubated with GST-HY5 protein and the DNA-protein complexes were separated on 6% native polyacrylamide gels. + or ++ indicate increasing amounts of unlabeled probes as the competitor.

**Figure 6:**
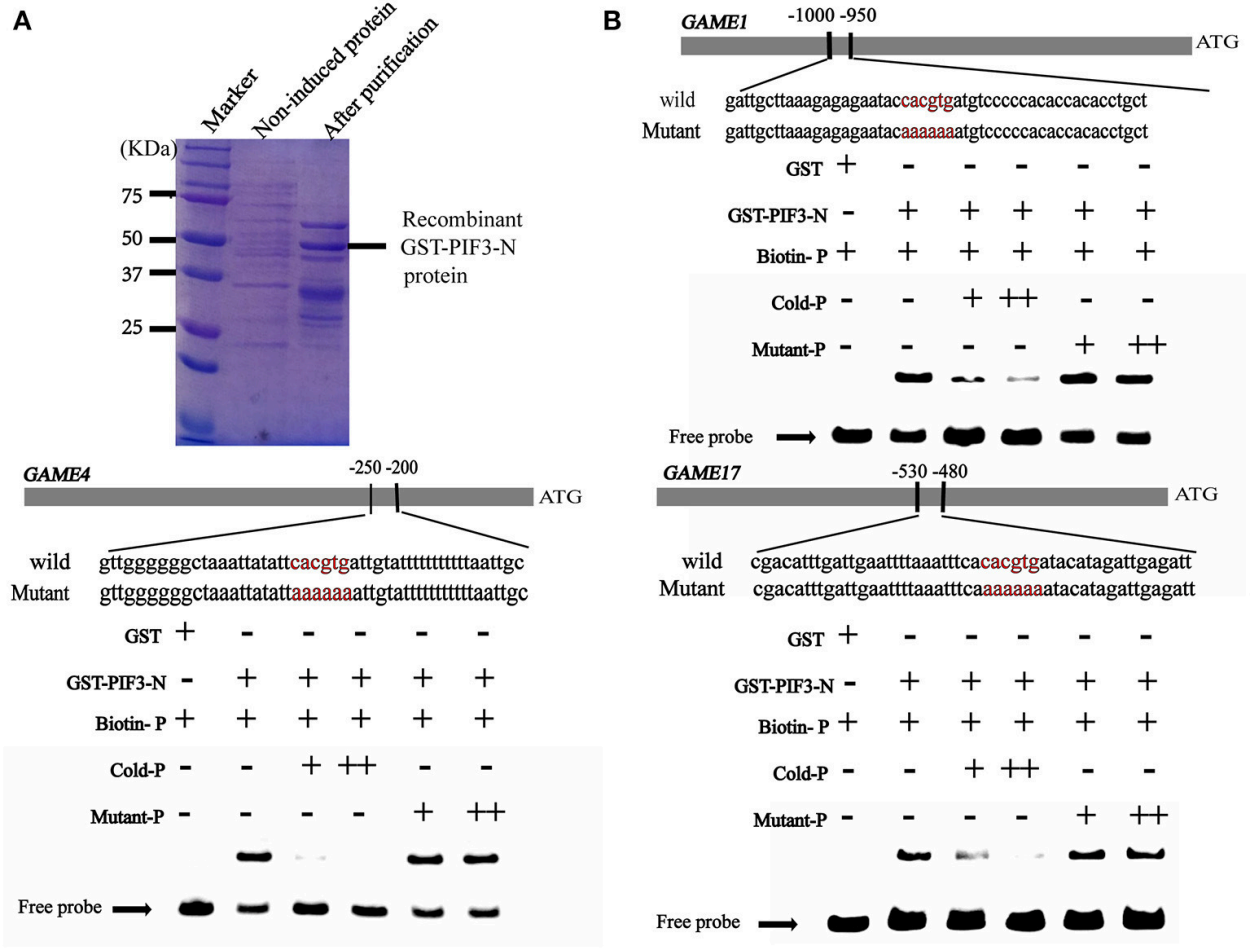
Electrophoretic mobility shift assay (EMSA) showing the association of *Sl*PIF3 with promoters of SGA biosynthetic genes *SlGAME1, SlGAME4*, and *SlGAME17*. **(A)** SDS–PAGE gel stained with Coomassie blue showing affinity purification of the recombinant *Sl*PIF3 protein used for the EMSA. **(B)** *Sl*PIF3 binds directly to the promoters of *SlGAME1, SlGAME4*, and *SlGAME17* containing G-box (CACGTG) elements. Biotin-labeled DNA probe from the promoters or mutant probes were incubated with GST-PIF3 protein and the DNA-protein complexes were separated on 6% native polyacrylamide gels. + or ++ indicate increasing amounts of unlabeled probes as the competitor.

It is well established that bZIP and bHLH TFs preferentially bind to the so-called G-box elements with a core sequence (CACGTG) in their target gene promoters. Sequence analysis identified G-box (CACGTG) elements in the promoters of SGA biosynthesis-related genes, including *GAME1/GAME4* and *GAME17* (Table S3). DNA electrophoretic mobility-shift assay (EMSA) was conducted to validate the interaction of HY5 and PIF3 with the *GAME1, GAME4, GAME17* promoters, using a DNA fragment containing the G-box elements in the promoter region of *GAME1, GAME4, GAME17* as probes (Figures 5, 6). Recombinant glutathione S-transferase (GST)-HY5 fusion protein and PIF3-GST recombinant protein were expressed and purified from *E. coli* (Figures 5, 6). The purified PIF3-GST recombinant protein contained multiple bands. and Western blot with anti-GST antibody showed these bands indeed included PIF3-GST protein (Figure S3). The recombinant HY5-GST and PIF3-GST were able to bind the *GAME1, GAME4*, and *GAME17* promoter fragments, respectively, and caused mobility shifts. However, they were not able to bind promoter fragments from *GAME2, GAME6, GAME11, GAME12*, and *GAME18* (Figure S1; Table S4). The binding was inhibited by increasing the amount of unlabeled competitor with the same sequence, but not by mutated probes (Figures 5, 6). In addition, the mobility shifts did not occur when the *GAME1, GAME4*, and *GAME17* promoter fragments were incubated with GST alone (Figures 5, 6), implying that HY5 and PIF3 transcription factors specifically bind to the promoters of *GAME1, GAME4*, and *GAME17*. ChIP-PCR was used in a further test to demonstrate that HY5 and PIF3 bind to the promoters of *GAME1, GAME4*, and *GAME17*.

**Figure 7:**
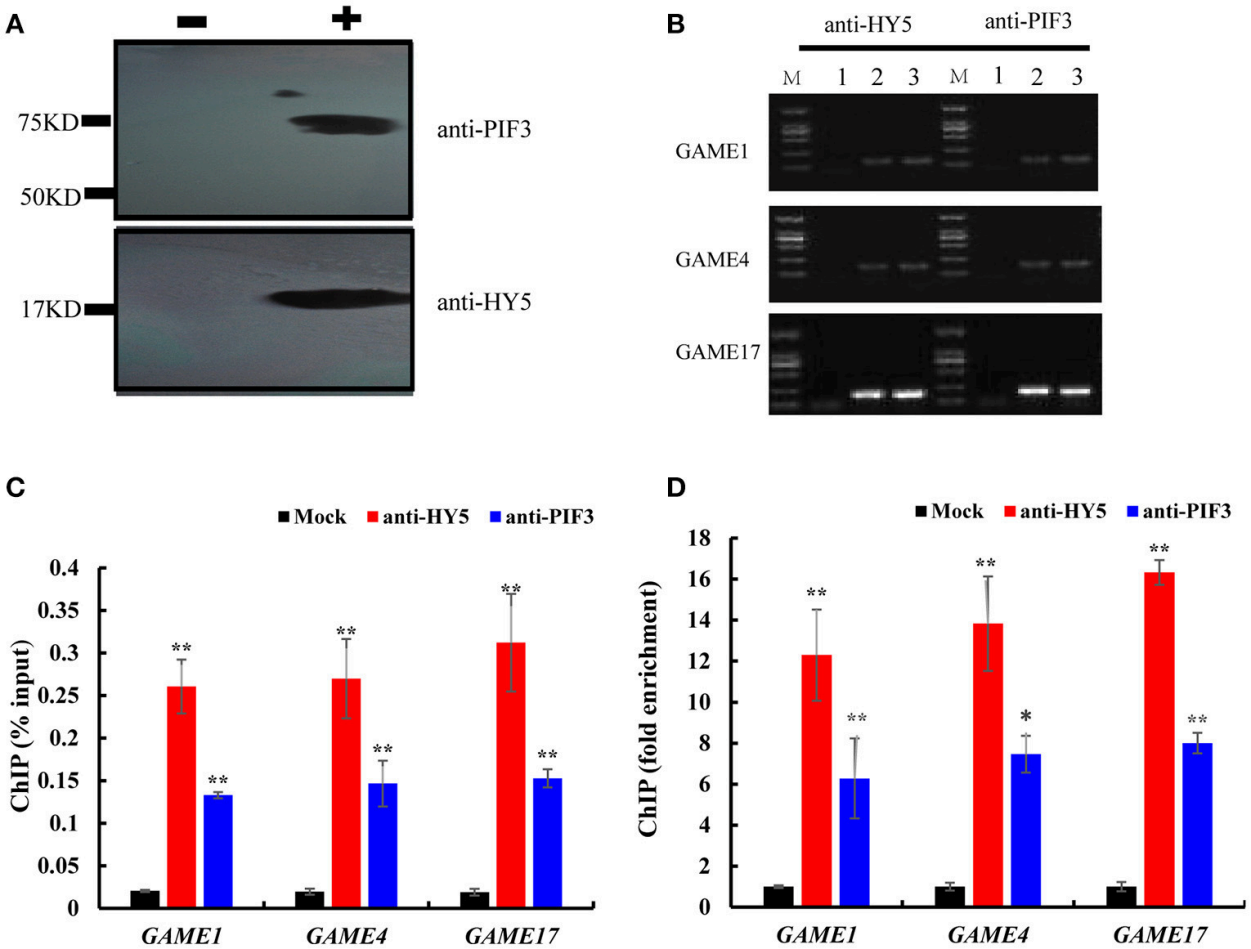
Chromatin immunoprecipitation reveals direct binding of HY5 and PIF3 to the promoters of genes involved in SGA metabolism. **(A)** Western blot to determine the quality of the polyclonal antibodies against plant proteins. **–** indicates negative serum; + indicates polyclonal anti-PIF3 and anti-HY5 respectively. **(B)** Agarose gel electrophoresis analysis of bound sequences through quantitative real-time PCR.
Chromatin extract precipitated without antibody (mock).Chromatin extract (input).ChIP with antibodies (anti-HY5 and anti-PIF3, respectively). Analyzed target genes are listed to the left of the corresponding images. **(C)** The input percentages derived for different antibodies. The columns represent the average input percentage from three independent experiments. **(D)** Quantification of ChIP analysis corresponding to images presented in **(C)**. Ordinates show the fold difference.

To investigate whether HY5 and PIF3 regulate the expression of these *GAME* genes by directly binding to their promoters *in vivo*, ChIP assay was performed to probe DNA-protein interactions within the natural chromatin. Western blots were used to determine the quality of the polyclonal antibodies against plant proteins (Figure 7A, Figure S2), with the input sample set as a positive control and the “no-antibody” (NoAb) is a negative control. Specific primers were designed for *GAME* to amplify promoter sequences surrounding G-box binding sites from the immunoprecipitated DNA (Table S2). The *actin* gene was used as internal control. Three independent immunoprecipitates were obtained under the same conditions and in all three samples the selected amplicons were found to be significantly enriched when compared to the negative control (Figure 7C). *GAME1, GAME4*, and *GAME17* were significantly enriched in HY5 (up to 12.29- to 16.33-fold) and PIF3 (up to 6.28- to 8.0-fold) samples.

**Figure 8:**
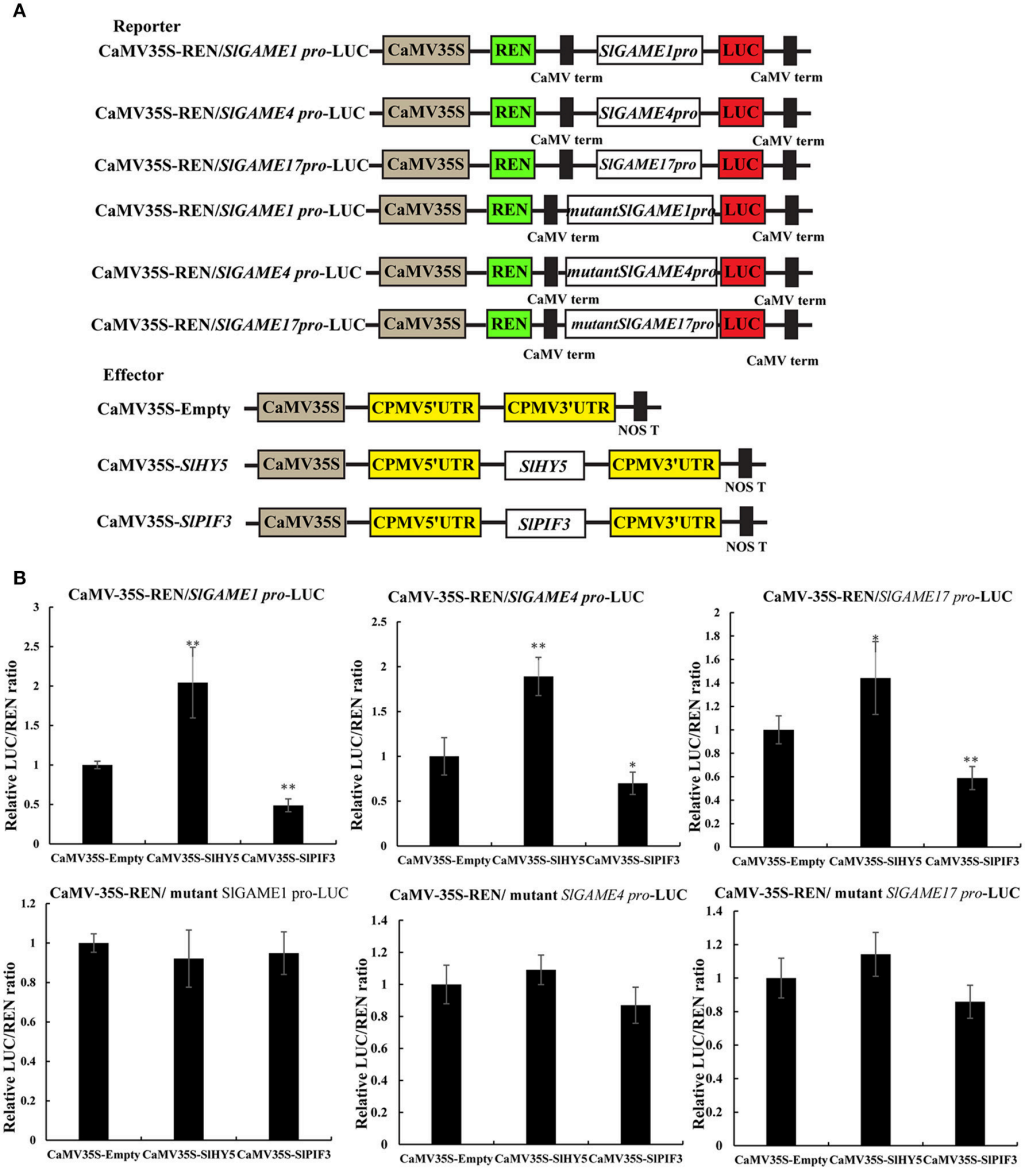
Transient expression analysis of the transcriptional activity of *SlPIF3* and *SlHY5*. **(A)** Schematic representation of the double reporters and effector plasmids used in the assay. **(B)** Transcriptional activation ability of *SlHY5* and transcriptional repression ability of *SlPIF3* in tomato leaves. Each value represents the means ± standard deviation (*n* = 6). Asterisks indicate significant differences in values (^*^P < 0.05; ^**^P < 0.01; Student's *t*-test).

Transient dual-luciferase reporter assays is a validated method that can show whether these G-box sequences are directly involved in changes in transcription mediated by light signal transduction TFs, by calculating as the transcriptional activity from the LUC/REN ratio (Hellens et al., 2005). Co-expression of *SlHY5* with *SlGAME1, SlGAME4*, or *SlGAME1*7 pro-LUC significantly increased the LUC/REN ratio (Figure 8B), suggesting that SlHY5 trans-activated SGAs biosynthetic genes. However, SlPIF3 suppressed the transcriptional activity of the *SlGAME1/4/17* promoters.

These data indicate that HY5 and PIF3 regulate SGA biosynthesis via transcriptional regulation of SGA biosynthesis-related genes, *GAME1, GAME4*, and *GAME17*.

## Discussion

### Transcriptional regulation of SGA biosynthesis genes

The steroidal alkaloids are among the best-known secondary metabolites in the Solanaceae family. At low concentrations in potato tubers, SGAs are unpalatable. However, at high concentrations, SGAs are harmful to mammals. To date, research on SGAs has focused mainly on the elucidation of their structure and composition in different plant species and analysis of their biosynthetic pathway (McCue et al., 2005, 2006, 2007; Shakya and Navarre, 2008; Itkin et al., 2011, 2013; Iijima et al., 2013; Cárdenas et al., 2015). Reports of TFs which are responsible for regulating the transcription of these *GAME* genes are scarce in tomato, but some APETALA2/Ethylene Response Factors (AP2/ERF) family members are considered the main transcriptional regulators. Cárdenas (Cárdenas et al., 2016) reported that GAME9 is a key regulator of SGA biosynthesis in tomato and acts additively with SlMYC2 in the activation of SGA biosynthetic genes. Interestingly, MYC2 has been reported to bind G-box *cis* binding elements in the promoters of target genes (Dombrecht et al., 2007; Cárdenas et al., 2016). Chonprakun found that a group of jasmonate-responsive transcription factors (JREs) of the ETHYLENE RESPONSE FACTOR (ERF) family also mediate transcriptional coordination of a series of metabolic SGA genes in tomato.(Chonprakun, 2016). HY5 directly binds to the G-box elements in the promoter regions of SGA genes (Figure 5). This finding is in agreement with previous reports of MYC2-recognizing elements present in similar regions of the targeted genes (Cárdenas et al., 2016) and also with the computational predictions of these elements in PIF3-regulated genes (Figure 6), suggesting a common feature for these transcription factors.

EMSA and ChIP-PCR both indicated that HY5 and PIF3 could directly bind to the SGA promoter regions (Figures 5–7) and transient dual-luciferase reporter assays showed that G-box sequences are directly involved in the light-stimulated changes in transcription (Figure 8). However, we cannot rule out the possibilities of a more indirect regulation, acting for example through metabolite-mediated feedback or involvement of other transcription factors. For example, GAME9, combined with transcription factor MYC2, could effectively transactivate the *HMGR1* promoter (Cárdenas et al., 2016). Furthermore, it seems reasonable to expect that HY5 and PIF3 regulate other SGA genes in cooperation with other TFs (Shoji and Hashimoto, 2011a,b; Moghe and Last, 2015).

### Impacts of altered light on SGA biosynthesis

In our study, we provide evidence to support the hypothesis that the expression of genes involved in the biosynthesis of SGAs is related to light (Figure 1) and that the TFs *SlHY5* and *SlPIF3* are involved in regulating SGAs biosynthesis. First, we showed that the expression of genes related to SGAs biosynthesis was down-regulated in *SlHY5*-silenced leaves and up-regulated in *SlPIF3*-silenced leaves (Figure 3). Second, we demonstrated that HY5 and PIF3 proteins bind to the promoters of *GAME1, GAME4, GAME17 in vitro* (Figures 5–8). Third, ChIP-PCR assays confirmed that HY5 and PIF3 proteins bind directly to their target genes and may regulate the expression of *GAME in vivo* (Figure 7). This raises thought-provoking questions regarding how to use light to reduce the toxicity of SGAs, thus improving the safety and nutritional value of Solanaceous species.

In previous studies, accumulation of glycoalkaloids was shown to be markedly influenced by light, with exposure to sodium and fluorescent light promoting higher glycoalkaloid accumulation compared to exposure to high and low-pressure mercury lights (Percival, 1999). Zrust found that the content of the two most important glycoalkaloids (alpha-chaconine and alpha-solanine) was increased in tubers of all 31 Czech potato varieties and three Slovakian varieties they examined when exposed to light. The reactions of potato varieties to lights of different quality and duration differed (Zrust et al., 2001). Light with different wavelengths has different effects on SGA accumulation. Red and blue light sources caused higher SGA accumulation in various potato cultivars (Wang et al., 2010). When Atlantic and Haryoung potato cultivars were exposed to seven different light qualities viz., purple, red, blue, green, yellow, UV, and fluorescent lights, Haryoung tuber accumulated 44% less SGA in yellow light compared to fluorescent light (Mekapogu et al., 2016). Although it is clear that light intensity, light color and the duration of light affect the biosynthesis of glycoalkaloids, the exact mechanisms underlying the observed differences in response are unclear.

### Light signal transduction-regulated SGA biosynthesis genes

HY5 has been shown to encode a basic leucine zipper TF required for light regulation of cell elongation, proliferation, and chloroplast development (Oyama et al., 1997). It has been found to bind G-box motifs and to promote activation of a number of light-induced genes, such as Rubisco small subunit (Ang et al., 1998; Chattopadhyay et al., 1998). It has also been shown that the G-box is over-represented in promoters of rhythmically co-expressed genes in the flavonol/anthocyanin metabolic pathway, suggesting that HY5 plays an essential role in plant secondary metabolism regulation through the G-box (Pan et al., 2009). Furthermore, HY5 integrates signals from photoreceptors to suppress the activity of the upstream MVA enzymes after illumination (Rodríguez-Concepción et al., 2004). In the cholesterol biosynthesis pathway, HY5 binds promoters of the 2C-methyl-D-erythritol 2,4-cyclodiphosphate synthase gene (At1g63970) in the upstream MEP pathway, two terpene synthases genes (At3g14520, At3g25830), and one geranylgeranyl pyrophosphate synthase gene (GGPS, At4g38460) (Lee et al., 2007). It appears that genes encoding enzymes in the mevalonate pathway, upstream of *GAME*, are also under some level of control by the HY5 TF. In our study, when HY5 was silenced *GAME* transcript levels decreased (Figure 3), indicating that HY5 acts as a positive regulator of SGA biosynthesis.

**Figure 9:**
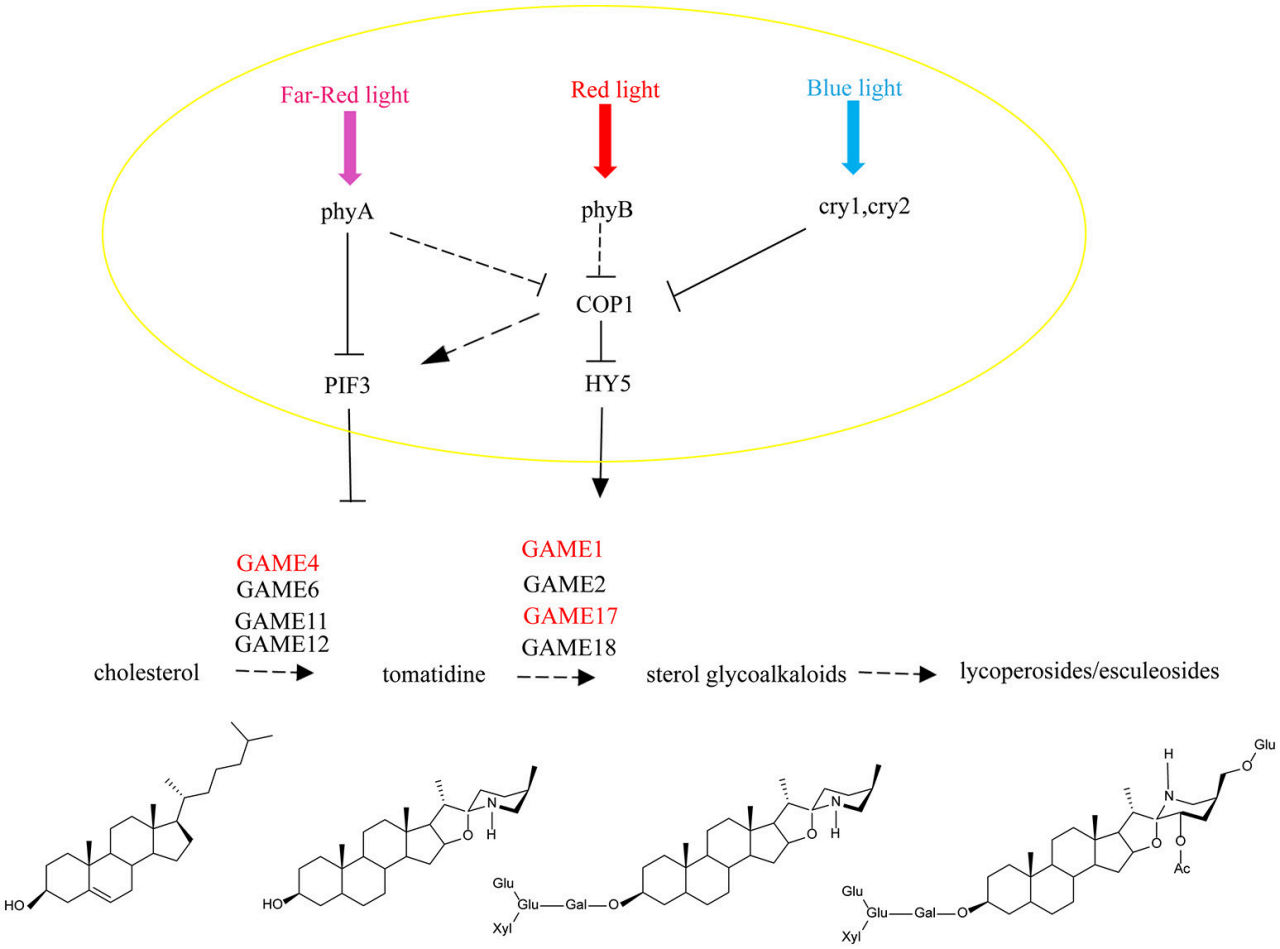
A model for light signal transmission control of the steroidal alkaloid pathway. HY5 activates the biosynthesis of SGAs in tomato and PIF3 represses gene expression across the biosynthesis pathway by independently binding to the *SlGAME1, SlGAME4*, and *SlGAME17* promoters.

HY5 promotes photomorphogenesis in the light whereas PIFs are bHLH transcription factors that are required for skotomorphogenesis in the dark and PIF3 binds to target genes and promotes skotomorphogenesis (Ni et al., 1998). As silencing of PIF3 increases the content of SGAs (Figure 3), our results, together with previous findings, suggest a significant role for light in the biosynthesis of SGA. On the basis of the biochemical and genetic data from these studies, a model for the involvement of light in SGA is proposed (Figure 9). In summary, HY5 regulates SGA biosynthetic genes *GAME1, GAME4*, and *GAME17* via binding to their promoters and activating transcription, however, PIF3 represses these genes. Our findings provide new insights into the transcriptional regulation of SGA biosynthesis.

It has been reported that light-induced PIF3 phosphorylation is a prerequisite for its degradation in the light (Al-Sady et al., 2006). PIF3 interacts with the G-box of *GAME* and other gene promoters, and inhibits their expression. After red light irradiation, PhyB is activated and moves from the cytoplasm into the nucleus to combine with PIF3 (Martínez-García et al., 2000) leading to its degradation, which releases the inhibition of *GAME* and other genes by PIF3.

Thus, HY5 and PIF3 control the biosynthesis of SGAs and this is not only limited to regulation of the *GAME* genes of the core pathway between cholesterol and a-tomatine, but also includes the upstream biosynthetic genes that convert acetyl-coenzyme A (acetyl-CoA) to cholesterol. This may be critical for ensuring the flux of precursors during the production of SGAs and to maintain the homeostasis in the interface between essential phytosterol biosynthesis and the cholesterol pathway.

To our knowledge, this is the first report of the regulation of expression of plant genes involved in SGAs biosynthesis by HY5 and PIF3 independently. Our results suggest a short signaling pathway from HY5/PIF3 to plant secondary metabolism by direct binding to the promoters of biosynthetic genes, which modulates *GAME* gene transcription. Understanding this light-induced process provides an opportunity for the generation of Solanaceous crops with modified levels of SGAs.

## Author contributions

CW conceived the project and wrote the article with contributions from all the authors. LM, DG, and YG provided technical assistance. DF supervised the experiments.

### Conflict of interest statement

The authors declare that the research was conducted in the absence of any commercial or financial relationships that could be construed as a potential conflict of interest.

## Supplementary material

Figure S1 Electrophoretic mobility-shift assays (EMSA) were conducted to validate the interaction of HY5 and PIF3 with the *GAME1, GAME4, GAME17* promoters (not with *GAME2, GAME6, GAME11, GAME12, GAME18*).

Figure S2 Western blot analysis of anti-HY5 and anti-PIF3 against plant proteins.

Figure S3 Immunoblot detection of PIF3-GST recombinant protein in GST-PIF3-purified protein showing many non-specific bands.

Table S1 List of primers used in VIGS. The first six primers were used for cloning *PDS, HY5* and *PIF3* from tomato into the pTRV2 vector. Other primers were used for qRT-PCR.

Table S2 List of primers used in EMSA, ChIP-PCR and DLR.

Table S3 The promoter region containing some *cis*-acting elements. ATG: initiation codon; The *cis*-acting element (CACGTG) is highlighted in red and the mutated nucleotides in the promoter sequences are AAAAAA.

Table S4 Biotin-labeled probes were used for EMSA. G box (CACGTG), CG hybrid (GACGTG), CA hybrid (GACGTA), and Z box (TACGTG) are known as HY5 and PIF3 consensus binding sequences.
