# Supplementary figures and images for: Manipulation of Light Signal Transduction Factors as a Means of Modifying Steroidal Glycoalkaloids Accumulation in Tomato Leaves
Source: Front Plant Sci. 2018 Apr 12;9:437. doi: 10.3389/fpls.2018.00437 (PMC5906708; doi:10.3389/fpls.2018.00437)

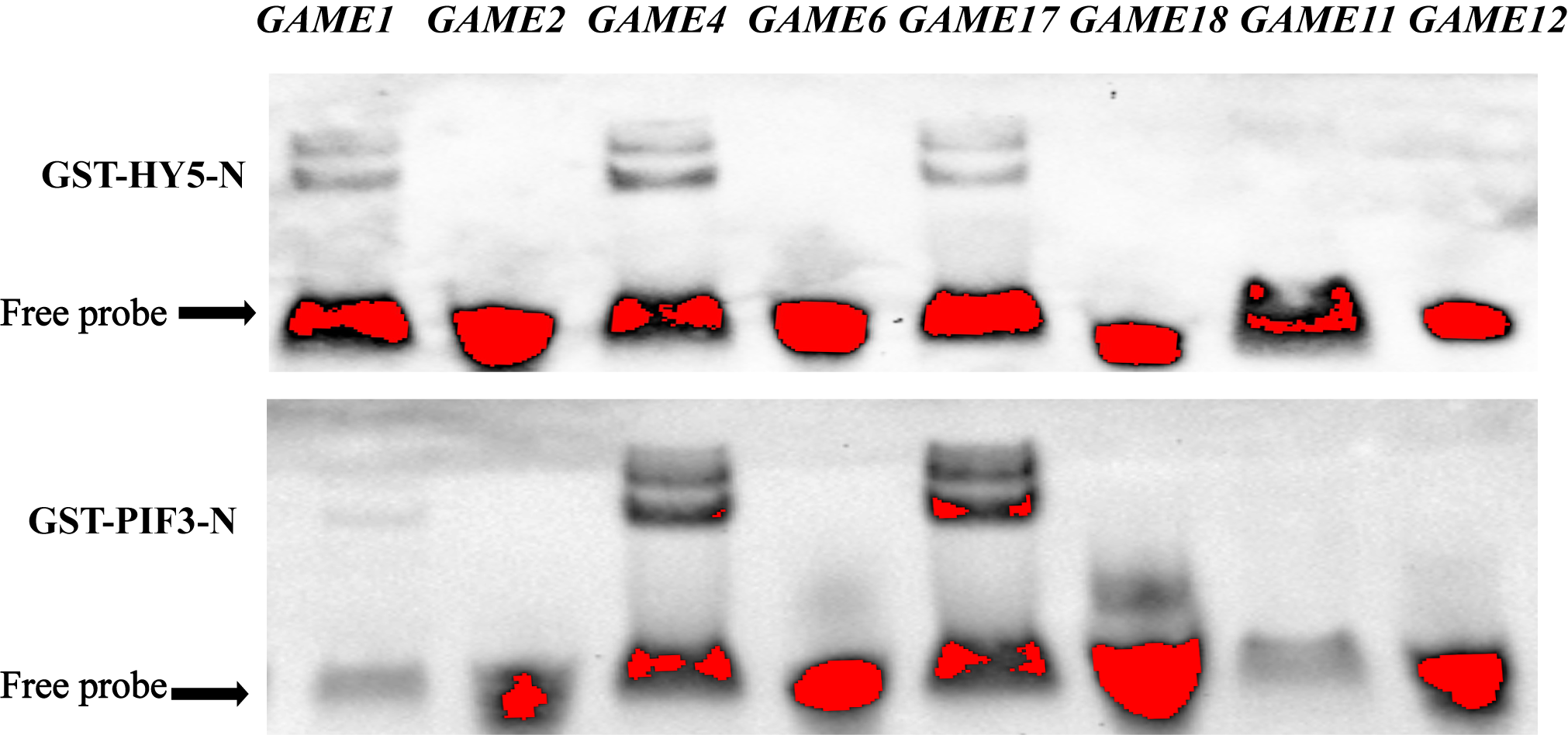

Supplement: Figure S1 — Electrophoretic mobility-shift assays (EMSA) were conducted to validate the interaction of HY5 and PIF3 with the GAME1, GAME4, GAME17 promoters (not with GAME2, GAME6, GAME11, GAME12, GAME18). [file Image1.TIF]

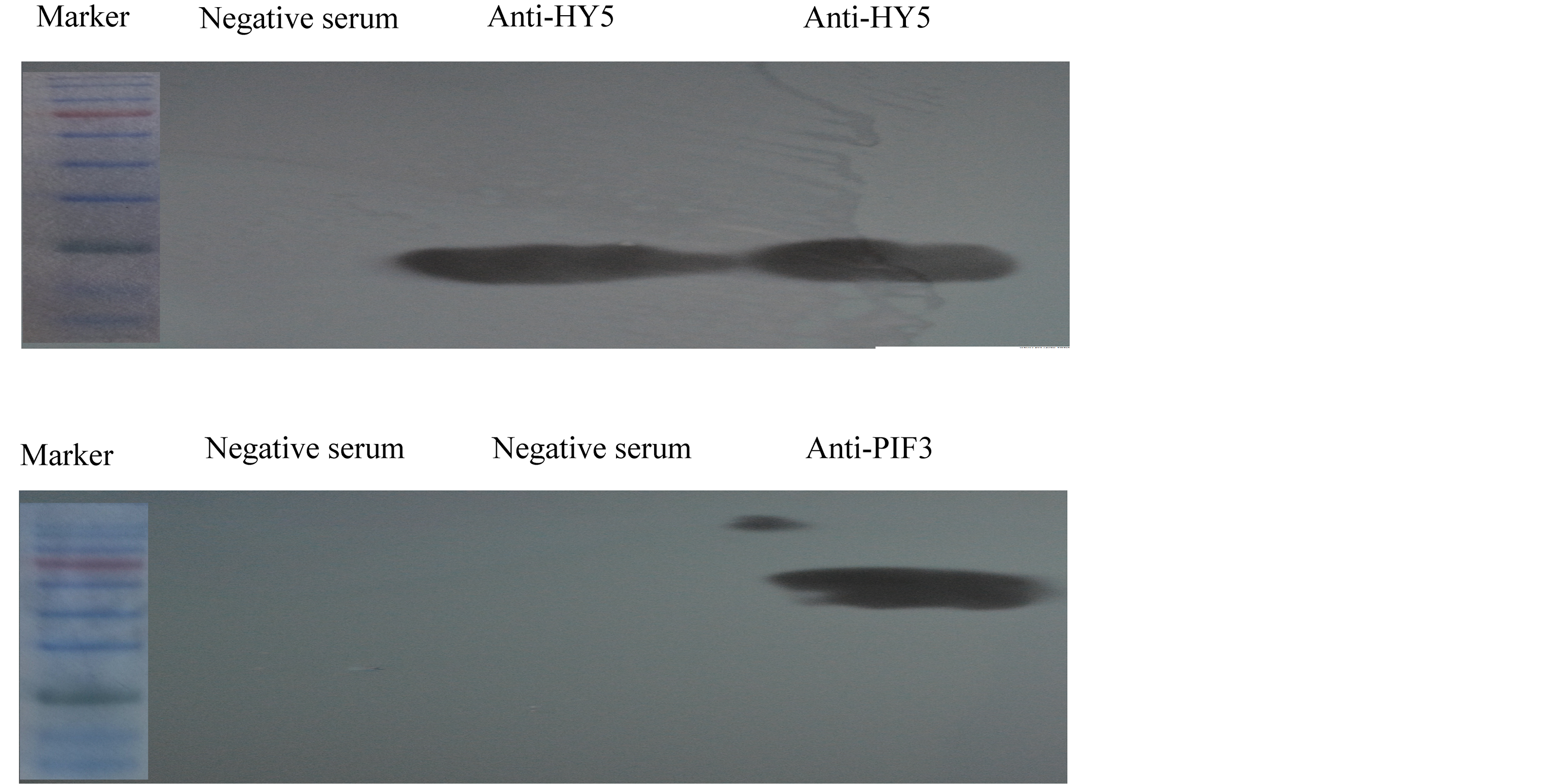

Supplement: Figure S2 — Western blot analysis of anti-HY5 and anti-PIF3 against plant proteins. [file Image2.TIF]

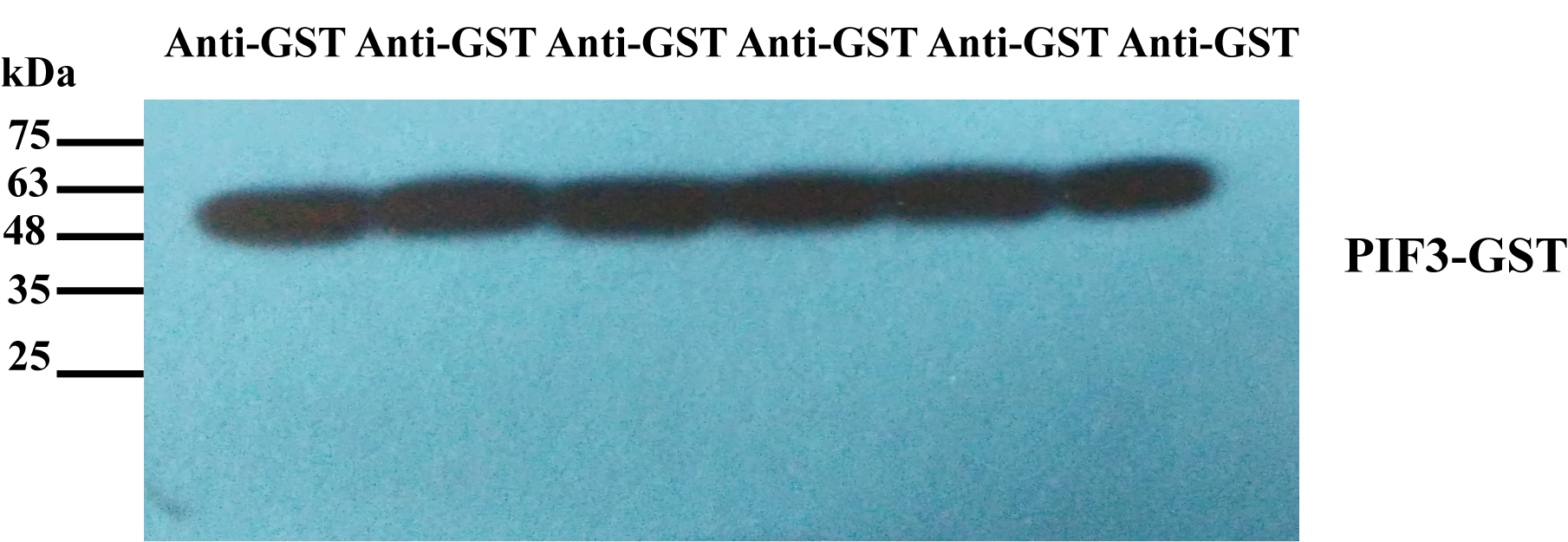

Supplement: Figure S3 — Immunoblot detection of PIF3-GST recombinant protein in GST-PIF3-purified protein showing many non-specific bands. [file Image3.TIF]
