# Supplementary material for: Manipulation of Light Signal Transduction Factors as a Means of Modifying Steroidal Glycoalkaloids Accumulation in Tomato Leaves
Source: Front Plant Sci. 2018 Apr 12;9:437. doi: 10.3389/fpls.2018.00437 (PMC5906708; doi:10.3389/fpls.2018.00437)
Supplement: Table S1 — List of primers used in VIGS. The first six primers were used for cloning PDS, HY5 and PIF3 from tomato into the pTRV2 vector. Other primers were used for qRT-PCR. [file Table1.DOC]

| Solyc IDs | Name | Sequence 5’-3’ |
| --- | --- | --- |
| Solyc03g123760 | TRV-PDS-F | CCGCTCGAGGACAGGGTGACAGATGAGGT |
|  | TRV-PDS-R | GGGGTACCTCTTTCCAGTCTTCAGGCA |
| Solyc08g061130 | TRV-HY5-F | CCGCTCGAGGGAAGAGATGGAGTATCGGC |
|  | TRV-HY5-R | GGGGTACCGCAACTTACCAAAGGCTGG |
| Solyc01g102300 | TRV-PIF3-F | GATCGCGAATTCTGAGTTTAGGGATGGGTATGG |
|  | TRV-PIF3-R | GATGGCGGATCCTGTCTGATTCTGTGGGCA |
| Solyc03g123760 | SlPDS-RT-F | AGATTGAGCTGAATGAGGATGGA |
|  | SlPDS-RT-R | AGTGAGCTTCTGCTGAAGAG |
| Solyc08g061130 | SlHY5-RT-F | CAAGCGACGAGTTCTATTGC |
|  | SlHY5-RT-R | ATCACTCTCCATACCTTCTTTGAG |
| Solyc01g102300 | SlPIF3-RT-F | CCTATGAATGTTGCAAGAGAAGG |
|  | SlPIF3-RT-R | CATTTGTTGCTTGTACCTGACTG |
| Solyc07g043490 | SlGAME1-RT-F | GCCTAATGAAGAAACAGCG |
|  | SlGAME1-RT-R | CGTTCCATACATCTATCCCG |
| Solyc07g043410 | SlGAME2-RT-F | GGAGCAAATGATTCGGGA |
|  | SlGAME2-RT-R | ATGTCCAGAGGCAAACCA |
| Solyc12g006460 | SlGAME4-RT-F | CAGTCGGTATTCCTTAGAGACA |
|  | SlGAME4-RT-R | TGCTCAACCCTGTAGTGAAG |
| Solyc07g043460 | SlGAME6-RT-F | GGAAGAACAGCAAGGGAAT |
|  | SlGAME6-RT-R | AACGAGAGAACCTGATGGATAG |
| Solyc07g043420 | SlGAME11-RT-F | GGCAGAGAATGCTTGTGAA |
|  | SlGAME11-RT-R | GTTCGTGACTCAGTTGTGTTTG |
| Solyc12g006470 | SlGAME12-RT-F | AAAGCGGAGGGTTCTTATG |
|  | SlGAME12-RT-R | ACTTCACCTGAGTGTCATTAGC |
| Solyc07g043480 | SlGAME17-RT-F | CCTCAAAGGCAAGACGAA |
|  | SlGAME17-RT-R | TCCACATCCTCAGGGTTGT |
| Solyc07g043500 | SlGAME18-RT-F | TTACCTGAACTTCCTCCTCATC |
|  | SlGAME18-RT-R | TGGCTGCGTTTACAGTGT |
| Solyc03g078400 | Actin-RT-F | CAGCAGATGTGGATCTCAAA |
|  | Actin-RT-R | CTGTGGACAATGGAAGGAC |
