# Supplementary material for: Manipulation of Light Signal Transduction Factors as a Means of Modifying Steroidal Glycoalkaloids Accumulation in Tomato Leaves
Source: Front Plant Sci. 2018 Apr 12;9:437. doi: 10.3389/fpls.2018.00437 (PMC5906708; doi:10.3389/fpls.2018.00437)
Supplement: Table S2 — List of primers used in EMSA, ChIP-PCR and DLR. [file Table2.DOC]

| Name | Sequence 5’-3’ |
| --- | --- |
| pGEX-PIF3-F | ccgcgtggatccccggaattcATGGGTGAGAGAAATGTTGAACATGG |
| pGEX-PIF3-R | gtcacgatgcggccgctcgagCTACAGAGATGCTCGAGGAATTGACA |
| pGEX-HY5-F | ccgcgtggatccccggaattcATGCAAGAGCAAGCGACGA |
| pGEX-HY5-R | gtcacgatgcggccgctcgagCTACTTCCTCCCTTCCTGTGCA |
| pET-PIF3-F | taagaaggagatatacatatgCAGTCTGGACAGAGTGTATTTGGTTT |
| pET-PIF3-R | gtggtggtggtggtgctcgagACCTCCTCGAGCAGCACAAG |
| ChIP-GAME1-F | TCTTACTGAAGTGTAGACCGCA |
| ChIP-GAME1-R | CGTTTCTTAGCAGGTGTGGT |
| ChIP-GAME4-F | GAAACCTTTTAACGAAAGTTGGGGG |
| ChIP-GAME4-R | GGCAACAATAATGATGAGACCAAATATGTG |
| ChIP-GAME17-F | TTCACACGTGATACATAGATTGAG |
| ChIP-GAME17-R | TCTATCTCAATTTATTCGACACATG |
| pEAQ-HY5-F | CAAATTCGCGACCGGTATGCAAGAGCAAGCGACGAGTTCTATTGCC |
| pEAQ-HY5-R | AGTTAAAGGCCTCGAGCTTCCTCCCTTCCTGTGCACCTGCT |
| pEAQ-PIF3-F | CAAATTCGCGACCGGTATGCCTCTCTCTGAGTTTTTGAAGATGGC |
| pEAQ-PIF3-R | AGTTAAAGGCCTCGAGCAAACTGGGACCAGCTTCATTTCCG |
| 0800-GAME1-F | TATAGGGCGAATTGGGTACCtttctccttccgcatggtgggagct |
| 0800-GAME1-R | TTGGCGTCTTCCATGGttttactaccccaatacaaagaaca |
| 0800-GAME4-F | TATAGGGCGAATTGGGTACCtagagttctattagtgcttgatcaag |
| 0800-GAME4-R | TTGGCGTCTTCCATGGggatggatttgtgaatttgattggagg |
| 0800-GAME17-F | TATAGGGCGAATTGGGTACCcaagtaaatgtgaaaagagaaaatgtaaggtag |
| 0800-GAME17-R | TTGGCGTCTTCCATGGgtgtgaagatgttgtggagtataac |
| pTRV1-F | TTACAGGTTATTTGGGCTAG |
| pTRV1-R | CCGGGTTCAATTCCTTATC |
| pTRV2-F | TGTTTGAGGGAAAAGTAGAGAACGT |
| pTRV2-R | TTACCGATCAATCAAGATCAGTCGA |
