# Supplementary material for: Manipulation of Light Signal Transduction Factors as a Means of Modifying Steroidal Glycoalkaloids Accumulation in Tomato Leaves
Source: Front Plant Sci. 2018 Apr 12;9:437. doi: 10.3389/fpls.2018.00437 (PMC5906708; doi:10.3389/fpls.2018.00437)
Supplement: Table S3 — The promoter region containing some cis-acting elements. ATG: initiation codon; The cis-acting element (CACGTG) is highlighted in red and the mutated nucleotides in the promoter sequences are AAAAAA. [file Table3.DOC]

*GAME1* Promoter

gattgcttaaagagagaataccacgtgatgtcccccacaccacacctgctaagaaacgagatctcaatgttaaaacttatagagataatgcacaagtaccccttcaatgtatgcgcgaaatctcagagacacacttatactattctaaggtcctattattcccctaaacttattttattaataattctctacccctttttgacctacttgacactattttgtgggtccaacgctgattaactatttttcaagctaatactatgtaggccgaaaaggggtagaaaattagttagaaaataagttcaggggggtaataggaccttagtatagcataaatgtgtctctggaatttcgggtatcggttgagggggtacttgtgcatttttccaaacttatatgatgaacttgtaatgcacatagagggaagtagaagctcgatatgaaccttacgactcagactcctttcccctacgatgagaatcgcgttgcaaccctttgtttggtggatttgggaaggaagttgaaatgagattgcaatttataatattttccatataatttttgaatatcaaaattttttacttaacatattgaatcaatgtaatctaatttaaattttaaaattaatcaaattaacttttgaaaaatatttccggaggggtggatcagaaagaaaaggtcttagtttcacaaaggataccaaagattaatgtcatttaattggatttaaaatacttcatgtgtctctttatttttaaattgacacacttattaagaaaacaattattatcatagtgaatttaccattttactcatattcttacgaagtgaatggattaaaaaattaagatttttttgtaaattcaaatgattaattaaagatatataatcaaggaagattaaactcgccattcattagtctataaatataaagatgagtaacagagcaaggtataagagttgttaaacaacaactgttctttgtattggggtagtaaaaATG

*GAME4* Promoter

atcttcacgtgaccaatgcatcaagtggaatttattaaaattaaatacgacaagatatgtagaaaattattgttacaaaaattagaaagaaacaaagtccatctttgaattctctcacataaaatattgggacccaacgttagaaacattattgttatggcaaaagaaaaaatcggttatcatcgacgtatctgatccagtagataagattatttctctcgttaatcgtaggttttgaatttgattttttagtattaaaaaaaatttttggtaagaaatgtctttccctatccttctaatggaatcctatgtagcgcgaattcgaaatagtctaatttcaatttaattattaaacacaaaatgaaaataaaaacaaaaatcctcattttactaataatcataataatgatgtagaaattattagctaatcccaaaatattaatcataaataaaatttaactaataatcaaaataaatattatatataaaccaacaatcaaaacaaaaactcatagtataagacagtacaacaagtgtaaaggttgtgtaaaagcttttaggtttctagtcaaataactatgatttttcttcaaaaatatcttaaagaattatatatataatcaatatatgatttttcattttttttggtttattattattttagtttttccctttcttttaattttttccataccttttgtccgtaatcatggaccatatcattatttgttaactgaaatgacaaaaataaaccaaaatttaacagctaacataaataagtttttctcaaaattcaacaacacatttaaaccttttcatttaaaaaaaaacaatttctttaataattttacaaaatcgaaaatatatttagtttatcaataatgaaaccttttaacgaaagttggggggctaaattatattcacgtgattgtatttttttttttaattgcacttctattaaggaataaaattataaagatttaactatctttgaaagtaaaaaggttagtattgtcttttcacatatttggtctcatcattattgttgccatatttttttagataaataaatgtagccatttactacctccaatcaaattcacaaatccatccaaattattcaaagaaaaaaaaatagagagagagaacaATG

*GAME17* Promoter

gactgcatgacacgtgataattaatttaaaatataattatatttatcttaaattttgctttatccttttcttttttattttttctcttactttttgactaattttcaattttttctttaattttaaatttaccttcccacttcacccatctcccacgtcagaactgacccgctcccctcattttatttctttttttcttcaactcccatactgcatataggtgtcactctctctacatgtgaacaaaccacacaagacgcaacatgaatttaactccttttcattatttattcttttcatttttcaagtatatatttggatgaaaatcatatcttataattaaaaatttatttattattttaaatcatcacacatgacttctatttaattaataaaaagatatgtattcactcaacacatccattcgccccccacaccccactcccagcacctatttaacatacagtacacccattatgcgcacgtgaaaaggtagtaaattcttcttcttcttctatataataaaaaataaaaactaaatttttaaaattttatttgtgtcatataaattgaaacagtgaaaataatgtatattattagtgacaataattaatgataaacgacatttgattgaattttaaatttcacacgtgatacatagattgagattattatttgacaattaaaatttaaattaacaaattacaataactgacaactaaatttttatttttattaaaagtttgattgaatttcaaaatttcatgtgtcgaataaattgagatagaaatagttatatatatatatgtatatcaattgaaaactattttagaagtattataaatcataatgattaatatataatttaaagtagttaaaatttaacatgattcaaaatacataaattaatggacttatttatgatattttcttctcaacacattgaagtatatatttggaaaaatgatgaatctaaattttaggctgtctgtttaaattattattttcatatgcaaacattaaaaatacggttgttggtattcggtagttgttacttcatatctatatatccttgtttagagtaacactccaataagtcttcaaaaaaaaaaaaaaatactaatagttatactccacaacatcttcacacATG
