# Supplementary material for: Manipulation of Light Signal Transduction Factors as a Means of Modifying Steroidal Glycoalkaloids Accumulation in Tomato Leaves
Source: Front Plant Sci. 2018 Apr 12;9:437. doi: 10.3389/fpls.2018.00437 (PMC5906708; doi:10.3389/fpls.2018.00437)
Supplement: Table S4 — Biotin-labeled probes were used for EMSA. G box (CACGTG), CG hybrid (GACGTG), CA hybrid (GACGTA), and Z box (TACGTG) are known as HY5 and PIF3 consensus binding sequences. [file Table4.DOC]

| Name | Probe sequence 5’-3’ |
| --- | --- |
| GAME2 | atcccaaaagagttgttag**gtcgtg**cacaaaacccgttttctgcgaaaat |
| GAME6 | ataataacaccgtttctctgcttta**agcgtg**ctttttatctcataactaaaaact |
| GAME11 | aagtaaattataaata**tacgtg**acgaaaacgatctttaaaattctttgta |
| GAME12 | gaaacgatataata**tacgtg**atacaagtttattaataaatcaatggccca |
| GAME18 | tgatacaactaaaagaca**tacgtt**taaaaaagcaaattgatcaagtcgtttg |
